# Performance of Point-of-Care Testing Compared with the Standard Laboratory Diagnostic Test in the Measurement of HbA1c in Indonesian Diabetic and Nondiabetic Subjects

**DOI:** 10.1155/2020/2037565

**Published:** 2020-07-09

**Authors:** Afiat Berbudi, Nofri Rahmadika, Adi Imam Tjahjadi, Rovina Ruslami

**Affiliations:** ^1^Department of Biomedical Sciences, Parasitology Division, Faculty of Medicine, Universitas Padjadjaran, Bandung, Indonesia; ^2^Infectious Disease Research Centre, Faculty of Medicine, Universitas Padjadjaran, Bandung, Indonesia; ^3^Department of Biomedical Sciences, Microbiology Division, Faculty of Medicine, Universitas Padjadjaran, Bandung, Indonesia; ^4^Department of Biomedical Sciences, Pharmacology and Therapy Division, Faculty of Medicine, Universitas Padjadjaran, Bandung, Indonesia

## Abstract

**Objective:**

This study is aimed at investigating if point-of-care testing for HbA1c (POCT-HbA1c) using the HemoCue® HbA1c 501 system could be an alternative method for diabetes screening and monitoring to replace the HbA1c measurement in a standard diagnostic laboratory.

**Design:**

This was a cross-sectional study to assess the agreement between POCT and a standard laboratory measurement method for determining the level of HbA1c. *Setting and Participants*. In total, 108 participants were recruited to participate in this study, consisting of 61 diabetics and 47 nondiabetics. The diabetic group comprised 37 females and 24 males, diagnosed with type 2 diabetes mellitus (DM) and undergoing diabetes treatment at several community health care centres in Bandung, West Java. The nondiabetic group consisted of 15 female and 32 male patients of several community health care centres and healthy volunteers. *Sample Collection and Analysis*. A venous blood sample was taken for routine HbA1c analysis by the diagnostic laboratory method. For the POCT-HbA1c, a blood sample was taken from the fingertip at the same time and analysed with the HemoCue® HbA1c 501 system. *Outcome Measures*. The HbA1c results of both methods were compared and analysed with a Bland-Altman agreement plot. The sensitivity and specificity of the POCT-HbA1c data were also compared with those of the standard diagnostic results.

**Results:**

Based on the Bland-Altman plot, the HbA1c level for 100 out of 108 (92.59%) subjects analysed by the POCT-HbA1c was within the range of the 95% limit of agreement. Compared with the standard diagnostic assay, the sensitivity of the POCT-HbA1c was 97.83% and its specificity was 77.42%.

**Conclusions:**

The high sensitivity and accuracy of POCT-HbA1c indicate that it is a potential method for diabetes screening and monitoring to replace the routine diagnostic laboratory HbA1c measurement, especially when a rapid result is required.

## 1. Introduction

Diabetes is a global health problem, indicated by a high blood glucose level caused by insufficient insulin production by the pancreas or decreasing insulin sensitivity [1]. Uncontrolled blood glucose in diabetes is associated with an increased risk of several complications, such as organ damage, cancer, dementia, and susceptibility to infection [2–8].

Early diagnosis of diabetes and associated complications provides an opportunity to commence timely effective preventive treatment that reduces the subsequent development or progression of macrovascular and microvascular disease. The key to preventing diabetic complications is by maintaining the blood glucose level within the normal range [9–11]. Consequently, measurements of fasting and 2-hour postprandial blood glucose are widely used in diabetes diagnosis and management [12]. However, the success of therapy and patient compliance is not well reflected by the blood glucose test at a specific time. Glucose bound with haemoglobin (glycated haemoglobin), known as HbA1c, is commonly used to evaluate blood glucose control over the previous 2–3 months [13, 14], with HbA1c levels higher than 6.5% indicating uncontrolled blood glucose level [15].

Information regarding the HbA1c level could help physicians to improve the management of diabetic patients through proper monitoring [16–19]. HbA1c testing has been included for diagnosing diabetes in the American Diabetes Association (ADA) guidelines since 2010 [20], and the World Health Organisation (WHO) also recommends HbA1c testing for diagnosing diabetes [21]. Similarly, recommendations to use HbA1c testing in diabetes diagnosis have been issued by the United Kingdom and New Zealand [22, 23], with the Australian Diabetes Society (ADS) expert committee also recommending HbA1c assessment to diagnose diabetes and be utilised for a corresponding Medicare Benefits Schedule (MBS) [24, 25]. HbA1c assessment benefits diabetic patients in several ways, including no requirement for fasting, less biological variability, and indicating an increased risk of cardiovascular disease (CVD) and retinopathy, compared to an oral glucose tolerance test (OGTT) or fasting glucose levels [26, 27]. Even though HbA1c assessment is important and beneficial in diabetes mellitus (DM) patient management, it is still not commonly used in Indonesia [28, 29] as it is costly and time-consuming.

Point-of-care testing (POCT) helps to ensure that after the diagnostic test, the patient receives the appropriate treatment in the hospital or the clinic [30]. Immediate results provided by POCT are important for reducing diabetes diagnosis time and initiating treatment for patients in remote areas [31]. Over the past few years, several medical device companies have produced rapid examination instruments for HbA1c, which generate the result in minutes, thus helping health workers, particularly doctors, to make rapid clinical decisions regarding treatment [32]. Nevertheless, the accuracy of the POCT-HbA1c measurement is still questionable [23, 33]; therefore, this study was conducted to compare the results of the POCT using the HemoCue® HbA1c 501 system with those of the standard laboratory method to evaluate whether this POCT could replace the standard laboratory methodology in Indonesia.

## 2. Materials and Methods

### 2.1. Research Subject and Sampling

The analysis was conducted by comparing the HbA1c level of diabetic and nondiabetic subjects obtained by the POCT-HbA1c with that of the standard diagnostic laboratory method. The diabetic group comprised 61 individuals from several community health care centres in Bandung that had been diagnosed with type 2 diabetes and were undergoing diabetes treatment. Nondiabetic subjects did not have diabetes symptoms and normal HbA1c and blood glucose levels. After giving informed consent, 5 ml of blood was collected to measure the HbA1c by the diagnostic laboratory test “Prodia” that has the National Glyco-Haemoglobin Standardisation Programme (NGSP) accreditation. The HbA1c level was analysed using high-performance liquid chromatography per WHO guidelines [12]. For the POCT-HbA1c, 5 *μ*l of blood was sampled from the fingertip at the same time and was analysed using the HemoCue® HbA1c 501 system (HemoCue AB, Sweden) certified by the International Federation of Clinical Chemistry and Laboratory Medicine (IFCC) and the NGSP according to the manufacturer's protocol. Briefly, the cartridge was inserted into the cartridge compartment. The reagent pack was prepared and applied to the blood specimen, then inserted into the cartridge, with the result automatically displayed after 5 minutes.

### 2.2. Analysis of Agreement Test

Bland-Altman plot statistical analysis was used to compare the HbA1c results obtained through the POCT using the HemoCue® HbA1c 501 system with those obtained by the standard diagnostic laboratory method.

### 2.3. Sensitivity and Specificity of POCT-HbA1c

The sensitivity and specificity of the POCT method using the HemoCue® HbA1c 501 system were compared with those of the standard laboratory method as a reference, using the standard cut-off HbA1c level of 6.5% [15].

### 2.4. Ethical Approval

All study participants were provided with the study information and gave written informed consent. All study procedures and methods were approved by the Ethical Committee of the Faculty of Medicine, Universitas Padjadjaran, Indonesia (No. 69/UN6.KEP/EC/2018).

## 3. Results

The mean value of HbA1c was 6.96% for the standard laboratory measurement versus 7.15% for POCT-HbA1c, with a mean difference of -0.187. A comparison of HbA1c measurements by the two methods is presented in Supplementary data 1.

### 3.1. Accuracy of HbA1c Measurement Using POCT-HbA1c

**Figure 1:**
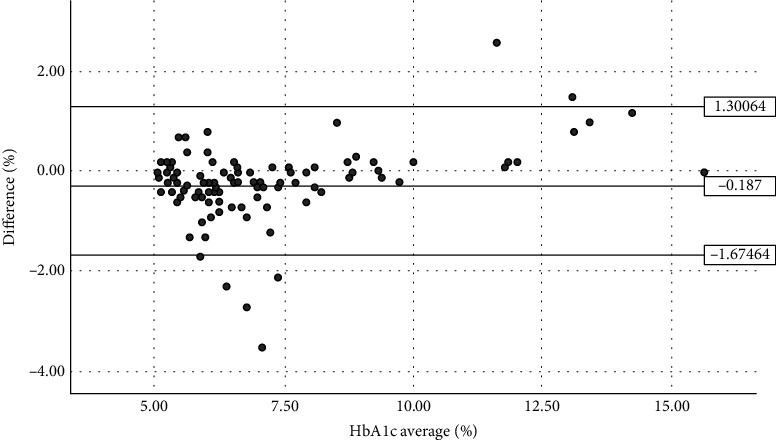
Analysis of the agreement between the POCT-HbA1c using the HemoCue HbA1c 501 system and standard diagnostic laboratory method (SD of bias = 0.759; 95% limits of agreement = −1.675–1.301; *n* = 108).

According to the Bland-Altman plot analysis, the HbA1c levels of 100 out of 108 subjects analysed with POCT-HbA1c and the standard diagnostic laboratory method were within the range of agreement limits (95% confidence intervals: -1.675 to 1.301), with only 3 upper outliers and 5 lower outliers outside the agreement limits range (Figure 1). This indicates that 92.59% of the HbA1c measurements by the HemoCue HbA1c 501 system are in line with the standard laboratory method.

### 3.2. POCT-HbA1c Using the HemoCue HbA1c 501 System Is Less Costly and More Rapid Compared to the Standard Diagnostic Laboratory HbA1c Method

A comparison of both methods in terms of unit cost, measurement time, required blood volume, and the analysis process is presented in Table 1. The POCT-HbA1c method is less costly, requiring fewer resources to rapidly measure HbA1c compared with the standard laboratory analysis. Furthermore, it only requires a small blood sample from the patient's fingertips, whereas the laboratory analysis requires a venous blood sample collected by a trained phlebotomist.

### 3.3. POCT-HbA1c Using the HemoCue® HbA1c 501 Demonstrates High Sensitivity Comparable to That of the Standard Laboratory Method

Based on the current guidelines, intensive therapy will be given to patients with an HbA1c level of more than 6.5%. To evaluate the clinical use of POCT-HbA1c, the HbA1c results were compared with those of the standard laboratory results using a cut-off point of 6.5% for HbA1c, as shown in Table 2. The 2 × 2 cross table reveals that the POCT-HbA1c has a sensitivity of 97.83%, while the specificity of this tool was 77.42%. The probability of having diabetes in a subject with HbA1c≥6.5 tested by POCT-HbA1c (positive predictive value) was 76.27%, while the probability of not having diabetes in a subject with HbA1c<6.5 tested by POCT-HbA1c (negative predictive value) was 97.96%. The diagnostic accuracy (effectiveness) of POCT-HbA1c expressed as a proportion of true positives and true negatives (correctly classified subjects by POCT-HbA1c) in all subjects was 86.11%.

## 4. Discussion

Fasting blood sugar level is the most commonly used indicator of diabetic patients' blood sugar control and compliance with treatment. Despite its effectiveness and low cost, fasting blood sugar level measurement is inconvenient since the patient must have fasted for 8 hours before blood collection. Hence, HbA1c measurement is used to predict the long-term complications of diabetes, with a high HbA1c closely related to an increased risk of CVD, nephropathy, and retinopathy; therefore, it could further predict the risk of mortality for diabetics [34]. The Centers for Disease Control and Prevention states that “every drop of HbA1c in the blood (for example, from 8.0% to 7.0%) could reduce microvascular complication risk to the eye, kidney, and nerve diseases up to 40%. Therefore, the HbA1c level is crucial for appropriate treatment” [32]. Currently, the ADA and the American Association of Clinical Endocrinologists recommend diabetics achieve an HbA1c level of 6.5% as an indicator of successful blood glucose control [15, 35].

POCT-HbA1c gives a more rapid result compared with laboratory-based HbA1c measurement, and the longer time gives the patient more opportunity to be noncompliant in checking their HbA1c levels [36, 37]. POCT-HbA1c makes it possible to monitor patients' prognosis and adherence to treatment in one visit [32]. Nevertheless, the controversy over the accuracy of POCT is still a concern for the users [38, 39]. Devices have been designed to accurately measure HbA1c, as it has become an important component in diabetes management [15], such as the HemoCue system. Such systems are advantageous, particularly for patients who have difficulties attending the laboratory for blood tests or repeat visits [40]. A study by Cagliero et al. showed that the HbA1c test result during the same visit was associated with increasing glycaemic control in diabetics [17]. It could also be an alternative to help with diagnosis, especially in areas where a laboratory is not available and is financially constrained or there is inadequate transportation to support referring patients to a more complete health infrastructure.

In this study, the mean difference between the POCT-HbA1c measurements and the standard lab was only -0.187, indicating that the POCT-HbA1c measurements were in line with the HbA1c measured by the standard diagnostic lab method. One hundred of 108 (92.59%) of the HbA1c measurements using the HemoCue® HbA1c 501 system were within the 95% limit of agreement, with only 8 (7.41%) outliers. The level of agreement between these results indicates that POCT using the HemoCue can be useful for monitoring blood glucose levels, supporting the clinician to manage the patient and improve the outcome of diabetes therapy.

Regarding the sensitivity and specificity, POCT-HbA1c using the HemoCue system was sensitive (92.83%) but less specific (specificity 77.42%) as 22.58% of nondiabetic subjects were identified as having an HbA1c≥6.5. Nonetheless, the proportion of positive results to population number (diagnostic accuracy) was high (86%).

Early detection of diabetes is important to prevent the disease progression and associated complications including coronary heart disease, stroke, cancer, or damage to various organs such as the kidneys, eyes, and nerves [27, 41]. Since screening is crucial for the early diagnosis of diabetes, the use of the sensitive HemoCue HbA1c system may contribute to preventing complications in diabetics. Furthermore, treatment in the early stage of diabetes can improve the outcome of diabetes management and reduce the risk of severe complications [42].

In addition to its potential for screening, POCT HbA1c using the HemoCue system can be a suitable alternative to monitoring diabetic patients, as it is less costly, rapid, and only requires a small finger prick blood sample compared to the standard laboratory method (Table 1), providing an immediate result [30]. Also, the POCT-HbA1c tool with instant results and affordable price could be an alternative method to increase patient compliance with diabetic medicine, thereby maintaining their blood glucose level in the normal range to improve the outcome of diabetes management [18].

## 5. Conclusions

POCT-HbA1c measurements using the HemoCue HbA1c system, compared to the standard laboratory method, are highly sensitive and accurate, as well as being less time-consuming and costly, with a more convenient blood collection. Therefore, this POCT system is a suitable tool for diabetes screening and management in the primary health care service, clinics, or remote areas with limited access to a laboratory. However, HbA1c measurement using the standard laboratory method is still recommended for the initial diabetes diagnosis.

## Figures and Tables

**Table 1:** A comparison of HbA1c measurement by POCT-HbA1c and the standard laboratory method.

Item	Laboratory test of HbA1c	POCT-HbA1c
Unit cost/sample (USD)^∗^	12.4	5.5
Time to get the result	2 days	5 minutes
Required blood volume	~1 ml	1 drop (~4 *μ*l)
Blood collection method	Phlebotomy	Finger prick
Sample processing	Laboratory	Bedside/clinic

^∗^Unit cost in Indonesia.

**Table 2:** Sensitivity and specificity of the POCT results using the HemoCue HbA1c 501 system based on a 6.5% cut-off point.

HbA1c test by POCT (%)	HbA1c test by standard laboratory method (%)	
	HbA1c≥6.5	HbA1c<6.5
HbA1c≥6.5	45	14
HbA1c<6.5	1	48
Total	46	62

^∗^The values in the table represent the number of subjects (*n* = 108). Sensitivity (45 out of 46/97.83%), specificity (48 out of 62/77.42%), positive predictive value (45 out of 59/76.27%), negative predictive value (48 out of 49/97.96%), false positive (14 of 62/22.58%), false negative (1 of 46/2.17%), and accuracy (86.11%).

## Data Availability

The data supporting the findings of this study are available within the supplementary material.

## Additional Points

*Strengths and Limitations of This Study*. (i) Our study shows that POCT using the HemoCue® HbA1c 501 system is sensitive but less specific compared to the standard laboratory diagnostic method. POCT-HbA1c makes it possible to monitor patient prognosis and compliance with the therapy regimen in one visit. (ii) Analysis of the agreement of the results from the two methods shows that the POCT-HbA1c can replace the more expensive and time-consuming standard lab method for use in the management of people with DM. (iii) This cross-sectional study had a limited number of participants.

## Supplementary Materials

Supplementary Materials Supplementary 1: comparison of HbA1c measured by diagnostic lab and POCT HemoCue.
