# Supplementary material for: Performance of Point-of-Care Testing Compared with the Standard Laboratory Diagnostic Test in the Measurement of HbA1c in Indonesian Diabetic and Nondiabetic Subjects
Source: J Diabetes Res. 2020 Jul 9;2020:2037565. doi: 10.1155/2020/2037565 (PMC7369652; doi:10.1155/2020/2037565)
Supplement: Supplementary Materials — Supplementary 1: comparison of HbA1c measured by diagnostic lab and POCT HemoCue. [file 2037565.f1.pdf]

Supplementary 1. Comparison of HbA1c measured by diagnostic lab and POCT HemaCue

No.	Subject ID	HbA1C (%)		Difference	Mean
		Standard Lab Assay	POCT		
1	1800001	9.3	9.4	-0.1	9.35
2	1800002	6.3	8.4	-2.1	7.35
3	1800003	6.6	6.6	0	6.6
4	1800004	6.4	5.6	0.8	6
5	1800005	7.6	8.2	-0.6	7.9
6	1800006	12.1	11.9	0.2	12
7	1800007	9.3	9.1	0.2	9.2
8	1800008	6	6.2	-0.2	6.1
9	1800009	6.4	6.5	-0.1	6.45
10	1800010	6.5	6.7	-0.2	6.6
11	1800011	8.8	8.6	0.2	8.7
12	1800012	6.5	6.7	-0.2	6.6
13	1800013	7.3	7.2	0.1	7.25
14	1800014	13.5	12.7	0.8	13.1
15	1800015	6.9	7.2	-0.3	7.05
16	1800017	6.8	7.1	-0.3	6.95
17	1800018	5.4	5.7	-0.3	5.55
18	1800019	6.5	6.7	-0.2	6.6
19	1800020	6.6	6.5	0.1	6.55
20	1800022	6.8	7	-0.2	6.9
21	1800023	5.6	6.1	-0.5	5.85
22	1800024	7.9	7.9	0	7.9
23	1800025	6.1	6.8	-0.7	6.45
24	1800026	6.8	6.8	0	6.8
25	1800027	6.6	6.4	0.2	6.5
26	1800029	5.9	6.1	-0.2	6
27	1800030	9	8.7	0.3	8.85
28	1800031	7.6	7.5	0.1	7.55
29	1800032	8.7	8.8	-0.1	8.75
30	1800033	6	6.3	-0.3	6.15
31	1800034	9	8	1	8.5
32	1800035	7.6	7.8	-0.2	7.7
33	1800036	6.5	6.7	-0.2	6.6
34	1800038	9.6	9.8	-0.2	9.7
35	1800039	8	8.4	-0.4	8.2
36	1800040	7.9	8.2	-0.3	8.05
37	1800041	6.3	6.3	0	6.3
38	1800043	6.4	6.6	-0.2	6.5

39	1800044	6.8	7.5	-0.7	7.15
40	1800045	7.3	7.5	-0.2	7.4
41	1800046	5.4	6.4	-1	5.9
42	1800047	7.6	7.6	0	7.6
43	1800048	8.5	6.9	1.6	7.7
44	1800049	5.8	6.2	-0.4	6
45	1800050	6.7	7.2	-0.5	6.95
46	1800051	6.9	7.1	-0.2	7
47	1800021	5.9	6.3	-0.4	6.1
48	1800028	8.1	8	0.1	8.05
49	1800056	7.2	7.5	-0.3	7.35
50	1800065	13.9	12.9	1	13.4
51	1800071	8.8	8.8	0	8.8
52	1800073	15.6	15.6	0	15.6
53	1800077	13.8	12.3	1.5	13.05
54	1800078	10.1	9.9	0.2	10
55	1800079	9.3	9.3	0	9.3
56	1800094	14.8	13.6	1.2	14.2
57	1800095	11.9	11.7	0.2	11.8
58	1800098	11.8	11.7	0.1	11.75
59	1800109	6.6	7.8	-1.2	7.2
60	1800110	6.3	7.2	-0.9	6.75
61	1800112	12.9	10.3	2.6	11.6
62	1800052	5.4	8.1	-2.7	6.75
63	1800053	5.6	6.1	-0.5	5.85
64	1800054	6.3	7	-0.7	6.65
65	1800055	6	6.4	-0.4	6.2
66	1800057	6.2	5.8	0.4	6
67	1800058	5.9	6.1	-0.2	6
68	1800059	5.2	5.7	-0.5	5.45
69	1800060	5.3	5.5	-0.2	5.4
70	1800061	5.9	6.5	-0.6	6.2
71	1800062	5.3	5.7	-0.4	5.5
72	1800066	5.6	6	-0.4	5.8
73	1800069	5.1	5.3	-0.2	5.2
74	1800070	5.5	6	-0.5	5.75
75	1800072	5.6	6.5	-0.9	6.05
76	1800074	5.8	6	-0.2	5.9
77	1800076	5.3	6.6	-1.3	5.95
78	1800080	6	6.2	-0.2	6.1
79	1800090	5.3	8.8	-3.5	7.05
80	1800096	5.8	5.9	-0.1	5.85
81	1800097	5.8	5.4	0.4	5.6
82	1800113	5.4	5.2	0.2	5.3
83	1800082	5.4	5.4	0	5.4
84	1800083	6	5.9	0.1	5.95
85	1800084	5.1	5.5	-0.4	5.3

86	1800085	4.9	5.3	-0.4	5.1
87	1800088	5.8	6.6	-0.8	6.2
88	1800089	5.7	6.3	-0.6	6
89	1800091	5.8	5.1	0.7	5.45
90	1800092	5.1	5.7	-0.6	5.4
91	1800099	5	6.7	-1.7	5.85
92	1800100	5.2	5.2	0	5.2
93	1800101	5.9	5.2	0.7	5.55
94	1800102	5.3	5.5	-0.2	5.4
95	1800103	5.3	5.1	0.2	5.2
96	1800104	5.3	5.2	0.1	5.25
97	1800105	5.2	7.5	-2.3	6.35
98	1800106	5	6.3	-1.3	5.65
99	1800108	6.2	6	0.2	6.1
100	1800116	4.9	5.3	-0.4	5.1
101	1800117	5	5	0	5
102	1800118	5	5.1	-0.1	5.05
103	1800119	5.3	5.1	0.2	5.2
104	1800120	5	5	0	5
105	1800121	5.3	5.4	-0.1	5.35
106	1800122	5.2	5	0.2	5.1
107	1800123	5.4	5.4	0	5.4
108	1800124	5.2	5	0.2	5.1
